# High miR‐202‐5p Expression at Initial Diagnosis is Associated With Tyrosine Kinase Inhibitor Resistance In Chronic Myeloid Leukemia—A Result From a Nested Case‐Control Study

**DOI:** 10.1002/jha2.70240

**Published:** 2026-02-12

**Authors:** Zi‐Yuan Nie, Jia Wang, Zi‐Yu Zhao, Ya‐Bei Zuo, Jin‐Ao Li, Tie‐Jun Gong

**Affiliations:** ^1^ Department of Hematology The Second Hospital of Hebei Medical University Shijiazhuang Hebei China; ^2^ Hospital Bossiness Management Office The Second Hospital of Hebei Medical University Shijiazhuang Hebei China; ^3^ Institute of Harbin Hematology & Oncology The First Hospital of Harbin Harbin Heilongjiang China

**Keywords:** biomarker, chronic myeloid leukemia, miR‐202‐5p, nested case‐control study

## Abstract

**Background:**

Tyrosine kinase inhibitor (TKI) resistance remains a critical challenge in chronic myeloid leukemia (CML). While mechanistic studies implicate miR‐202‐5p in resistance, its clinical relevance as a biomarker at diagnosis requires validation.

**Methods:**

A nested case‐control design was employed within a prospective cohort of 797 newly diagnosed chronic‐phase CML patients. Of these, 31 patients who developed TKI resistance (per ELN 2020 criteria, without ABL mutations) were matched 1:4 to 124 TKI‐sensitive controls on age, sex, Sokal score, and baseline white blood cell count. miR‐202‐5p expression was quantified by qRT‐PCR from diagnostic peripheral blood mononuclear cells (PBMCs). Statistical analyses included conditional logistic regression and receiver operating characteristic (ROC) curve analysis.

**Results:**

The expression level of miR‐202‐5p was significantly elevated in the TKI‐resistant group (1.68 ± 0.45) compared to the TKI‐sensitive group (1.26 ± 0.32) (*p* < 0.001). Conditional logistic regression analysis revealed that elevated miR‐202‐5p expression was strongly correlated with an increased risk of TKI resistance (OR = 15.21, 95% CI: 4.87–47.51; *p* < 0.001). ROC curve analysis demonstrated that miR‐202‐5p had moderate diagnostic accuracy for identifying TKI resistance (AUC = 0.73, 95% CI: 0.65–0.81). Using the optimal cut‐off value of 1.63 determined by the Youden Index, the proportion of TKI resistance was significantly higher in the high‐expression group (61.29% vs. 12.10%, *p* < 0.001).

**Conclusion:**

Elevated miR‐202‐5p expression at diagnosis is significantly associated with TKI resistance in CML. These findings support its potential as a clinical biomarker for identifying high‐risk patients, which could aid in early risk stratification and guide therapeutic strategy.

**Trial Registration:**

The authors have confirmed clinical trial registration is not needed for this submission

1

Dear editor:

The advent of tyrosine kinase inhibitors (TKIs) targeting the BCR‐ABL1 oncoprotein has fundamentally altered the prognosis of chronic myeloid leukemia (CML), transforming it into a manageable chronic condition for most patients. Despite this success, resistance to TKIs remains a significant clinical hurdle, affecting approximately 20%–30% of patients and leading to suboptimal responses, disease progression, and poorer outcomes [1]. While the development of new‐generation TKIs has broadened therapeutic options, resistance driven by both BCR‐ABL1‐dependent mechanisms and, critically, BCR‐ABL1‐independent pathways involving aberrant activation of survival signals persists [2, 3, 4]. Early identification of patients at high risk of resistance is paramount for implementing proactive strategies, such as closer monitoring, early switch to alternative TKIs, or consideration of novel combination therapies. However, current predictive tools beyond BCR‐ABL1 mutation screening and clinical risk scores remain limited and often fail to capture the complexity of intrinsic resistance mechanisms.

MicroRNAs (miRNAs), small non‐coding RNAs that fine‐tune gene expression, have emerged as crucial regulators of CML pathogenesis and therapy response. Dysregulation of specific miRNAs can influence key processes like apoptosis evasion, drug efflux, and survival pathway activation, contributing significantly to TKI resistance [5, 6]. Our prior mechanistic work identified miR‐202‐5p as a pivotal player in this context, demonstrating its role in suppressing apoptosis via the STAT5A/miR‐202‐5p/USP15/Caspase‐6 regulatory axis [7]. However, the translation of miR‐202‐5p dysregulation into a clinically applicable biomarker for TKI resistance at diagnosis required validation. To address this gap, we leveraged the strengths of a nested case‐control study design within a prospective cohort. This robust epidemiological approach minimizes selection bias, efficiently utilizes resources from an existing cohort, and allows for rigorous matching on potential confounders, making it particularly well‐suited for evaluating biomarkers for relatively rare outcomes like TKI resistance.

This study employed a nested case‐control design within a prospective cohort of 797 newly diagnosed chronic‐phase CML patients initiated on TKI therapy between 2020 and 2023. From this parent cohort, we identified all patients who developed TKI resistance without detectable ABL kinase mutations (cases). TKI resistance was strictly defined per European LeukemiaNet (ELN) 2020 guidelines as failure to achieve complete hematologic response (CHR) by 3 months, BCR‐ABL1 transcript level (IS) > 10% at 6 months, or failure to achieve major molecular response (MMR, BCR‐ABL1 IS ≤ 0.1%) by 12 months. For each case, up to four TKI‐sensitive controls were selected from the cohort using incidence density sampling. Controls were individually matched to cases on age, sex, Sokal risk score, and baseline white blood cell count. This process yielded a final analytical sample of 31 cases and 124 controls (total *n* = 155). The study was approved by the Ethics Committee of The First Hospital of Harbin and the Ethics Committee of The Second Hospital of Hebei Medical University.

Peripheral blood mononuclear cells (PBMCs) collected at diagnosis underwent miR‐202‐5p quantification via qRT‐PCR (U6 normalization, 2^ΔCt^ method). Descriptive statistics compared groups (*t*‐tests, chi‐square). Given the 1:4 matched design, analyses accounted for the matched‐set structure. Matching effectiveness was confirmed by comparing the distribution of matched variables (age, sex, Sokal score, and baseline WBC) within sets. The association between miR‐202‐5p expression and TKI resistance was analyzed using conditional logistic regression, with miR‐202‐5p treated as both a continuous variable. Odds ratios (ORs) with 95% confidence intervals (CIs) were calculated. The discriminatory ability of continuous miR‐202‐5p was evaluated by ROC curve analysis. Analyses used R version 4.3.0, with a two‐sided *p* < 0.05 considered significant.

The final matched study population consisted of 31 TKI‐resistant cases and 124 TKI‐sensitive controls. The baseline characteristics of the groups are presented in Table 1. As a result of the 1:4 individual matching on age, sex, Sokal risk score, and baseline WBC count, the distributions of these variables were closely balanced between cases and controls.

**TABLE 1:** Baseline characteristics of the study population.

Characteristic	Resistant group (*n* = 31)	Sensitive group (*n* = 124)
Age (years)	45.65 ± 13.26	45.83 ± 10.55
Sex (male/female)	18/13	73/51
Sokal score (high/intermediate/low)	12/10/9	44/44/36
Baseline WBC (×10^9^/L)	124.64 ± 29.79	130.158 ± 29.12

*Note*: Cases and controls were individually matched 1:4 on all variables listed in this table. Data are presented as mean ± standard deviation or number. Given the matched design, formal statistical comparisons between groups for these matched variables are not appropriate and are therefore not provided; the similarity in distributions demonstrates the effectiveness of the matching process.

Abbreviations: TKI, tyrosine kinase inhibitor; WBC, white blood cell count.

Analysis of miR‐202‐5p expression revealed a highly significant difference between groups. The mean relative expression level (± SD) was 1.68 ± 0.45 in the TKI‐resistant group compared to 1.26 ± 0.32 in the TKI‐sensitive group (*p *< 0.001;Figure S1). When analyzed as a continuous variable using conditional logistic regression, each unit increase in miR‐202‐5p expression was strongly associated with TKI resistance (OR = 15.21, 95% CI: 4.87–47.51; *p* < 0.001). ROC curve analysis further evaluated the predictive utility of miR‐202‐5p expression. The AUC was 0.73 (95% CI: 0.65–0.81), indicating moderate discriminatory ability (Figure 1). ROC curve analysis determined the optimal discriminatory cutoff for miR‐202‐5p expression to be 1.63 (corresponding to a Youden Index = 0.492), the sensitivity for predicting resistance was 61.3%, and the specificity was 87.9%. Using the Youden Index, we set the cutoff value for miR‐202‐5p expression at 1.63 and accordingly divided the patients into high‐expression and low‐expression groups. The analysis revealed that the proportion of TKI resistance was significantly higher in the high‐expression group compared to the low‐expression group (61.29% vs. 12.10%, *p* < 0.001). (Table S1)

**FIGURE 1:**
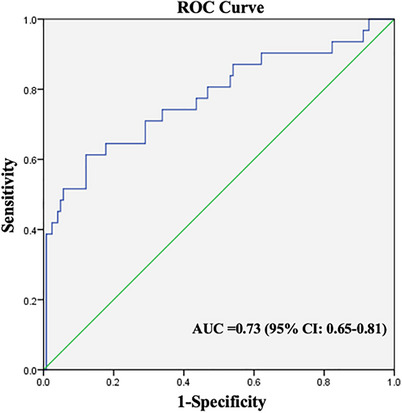
Receiver operating characteristic (ROC) curve analysis of miR‐202‐5p expression for predicting TKI resistance. The ROC curve depicts the predictive performance of baseline miR‐202‐5p expression levels (measured by qRT‐PCR in PBMCs) for the development of TKI resistance in 155 chronic‐phase CML patients. The area under the curve (AUC) is 0.73 (95% CI: 0.65–0.81). (AUC = 0.5, no discrimination).

Dysregulation of specific miRNAs has been implicated in the development of TKI resistance, making them attractive candidates for both prediction and therapeutic targeting. However, the ability of miRNA to predict TKI resistance at diagnosis is limited. The nested case‐control design employed in this study offers distinct advantages for investigating biomarkers of relatively rare outcomes like TKI resistance. This efficient approach, embedded within a prospective cohort, minimizes selection bias, ensures pre‐treatment sample availability, and enables rigorous confounder matching [8]. The utility of this design is well‐established, as demonstrated by its successful application in identifying predictive markers for antibiotic resistance in bacterial infections [9] and antiretroviral resistance in HIV [10]. To our knowledge, this represents the first application specifically for identifying TKI resistance biomarkers in CML. Critically, the strong association observed between elevated diagnostic miR‐202‐5p expression and resistance provides clinical validation of prior mechanistic work establishing miR‐202‐5p's role in suppressing apoptosis via the STAT5A/miR‐202‐5p/USP15/Caspase‐6 axis. First, the number of resistant cases (*n* = 31) is relatively small, limiting statistical power for subgroup analyses and potentially affecting the precision of the effect estimate (wide CI). Larger, multicenter validation is essential. Second, establishing a clinically robust cutoff threshold for miR‐202‐5p expression remains challenging; our median‐based cutoff requires further refinement and prospective confirmation in independent cohorts to define an optimal value.

Despite these limitations, miR‐202‐5p shows significant potential as a biomarker for TKI resistance. Its assessment at diagnosis could enhance risk stratification, allowing for closer monitoring or consideration of alternative strategies for high‐risk patients. Importantly, our prior in vitro work demonstrated that pimozide can overcome miR‐202‐5p‐mediated TKI resistance, suggesting a potential therapeutic strategy for patients identified by this biomarker. Integrating miR‐202‐5p testing into clinical practice could guide more personalized treatment approaches aimed at preventing or overcoming resistance in CML.

## Funding

This work was supported National Natural Science Foundation of Hebei Province (H2023206153).

## Ethics Statement

The study was approved by the Ethics Committee of The First Hospital of Harbin and the Ethics Committee of The Second Hospital of Hebei Medical University.

## Conflicts of Interest

The authors declare no conflicts of interest.

## Supporting information




**Figure S1**: miR‐202‐5p expression in TKI‐resistant versus TKI‐sensitive CML patients at diagnosis. Relative miR‐202‐5p expression levels in peripheral blood mononuclear cells (PBMCs) measured by quantitative RT‐PCR for 31 tyrosine kinase inhibitor (TKI)‐resistant patients and 124 TKI‐sensitive patients. Horizontal bars represent group means. Normalized to U6. ***P < 0.001 vs. TKI‐sensitive group. **Table S1**: miR‐202‐5p Expression between TKI‐Resistant and TKI‐Sensitive Patients.

## Data Availability

The data used or analyzed during the current study are available from the corresponding author on reasonable request.
