# Supplementary material for: High miR‐202‐5p Expression at Initial Diagnosis is Associated With Tyrosine Kinase Inhibitor Resistance In Chronic Myeloid Leukemia—A Result From a Nested Case‐Control Study
Source: EJHaem. 2026 Feb 12;7(1):e70240. doi: 10.1002/jha2.70240 (PMC12896366; doi:10.1002/jha2.70240)
Supplement: Supplementary file 1 — Figure S1: miR‐202‐5p expression in TKI‐resistant versus TKI‐sensitive CML patients at diagnosis. Relative miR‐202‐5p expression levels in peripheral blood mononuclear cells (PBMCs) measured by quantitative RT‐PCR for 31 tyrosine kinase inhibitor (TKI)‐resistant patients and 124 TKI‐sensitive patients. Horizontal bars represent group means. Normalized to U6. ***P < 0.001 vs. TKI‐sensitive group. Table S1: miR‐202‐5p Expression between TKI‐Resistant and TKI‐Sensitive Patients. [file JHA2-7-e70240-s001.docx]

**Supplementary Figure 1**


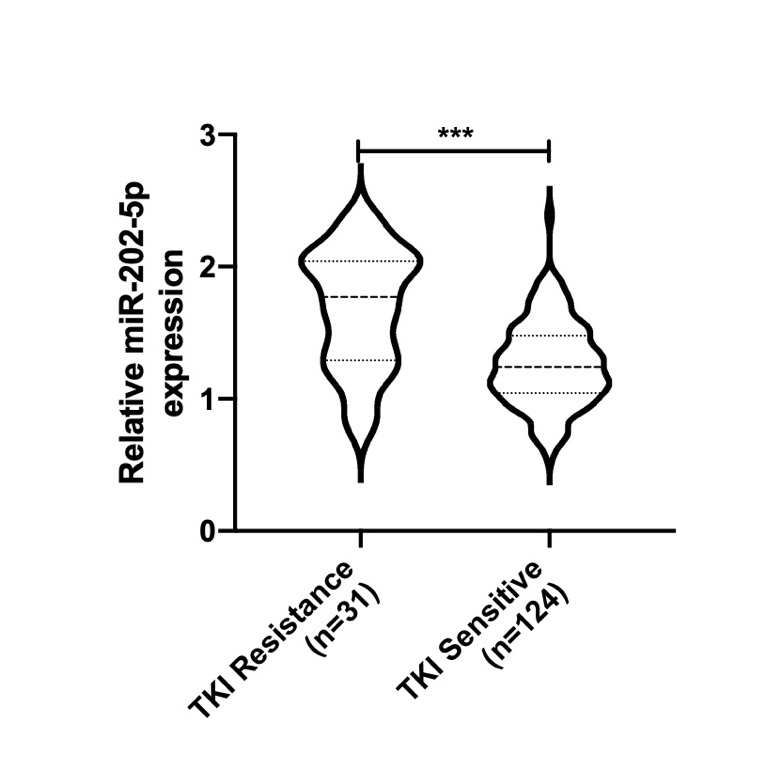


**Supplementary Figure 1. miR-202-5p expression in TKI-resistant versus TKI-sensitive CML patients at diagnosis.** Relative miR-202-5p expression levels in peripheral blood mononuclear cells (PBMCs) measured by quantitative RT-PCR for 31 tyrosine kinase inhibitor (TKI)-resistant patients and 124 TKI-sensitive patients. Horizontal bars represent group means. Normalized to U6. ****P* < 0.001 vs. TKI-sensitive group.

**Supplementary Table 1. miR-202-5p Expression between TKI-Resistant and TKI-Sensitive Patients**

| **Characteristic** | **Resistant Group (n=31)** | **Sensitive Group (n=124)** | ***χ^2^*** | **P-value** |
| --- | --- | --- | --- | --- |
| miR-202-5p expression (low/high) | 12/19 | 109/15 | 77.875 | ＜0.001 |
